# Ongoing Initiatives to Improve the Quality and Efficiency of Medicine Use within the Public Healthcare System in South Africa; A Preliminary Study

**DOI:** 10.3389/fphar.2017.00751

**Published:** 2017-11-09

**Authors:** Johanna C. Meyer, Natalie Schellack, Jacobus Stokes, Ruth Lancaster, Helecine Zeeman, Douglas Defty, Brian Godman, Gavin Steel

**Affiliations:** ^1^School of Pharmacy, Sefako Makgatho Health Sciences University, Garankuwa, South Africa; ^2^Contract Management, South Africa Directorate: Affordable Medicines, Sector Wide Procurement, National Department of Health, Pretoria, South Africa; ^3^Essential Drugs Programme, South Africa Directorate: Affordable Medicines, Sector Wide Procurement, National Department of Health, Pretoria, South Africa; ^4^Health Systems Trust, Pretoria, South Africa; ^5^Mediclinic Southern Africa, Stellenbosch, South Africa; ^6^Strathclyde Institute of Pharmacy and Biomedical Sciences, University of Strathclyde, Glasgow, United Kingdom; ^7^Division of Clinical Pharmacology, Karolinska Institute, Karolinska University Hospital, Stockholm, Sweden; ^8^Health Economics Centre, Management School, University of Liverpool, Liverpool, United Kingdom; ^9^Sector Wide Procurement, National Department of Health, Pretoria, South Africa

**Keywords:** essential medicines, health care reforms, pharmacovigilance, procurement, South Africa, supply chain

## Abstract

**Introduction:** South Africa has an appreciable burden of both communicable and non-communicable diseases as well as high maternal, neonatal, and child morbidity. In recent years there have been significant strides with improving the public health system, and addressing current inequalities, with the right to health a constitutional provision in South Africa. Initiatives include the introduction of National Health Insurance, programmes to enhance access to medicines for patients with chronic diseases, as well as activities to improve care in hospitals, including improving pharmacovigilance. Consequently, the objective of this paper is to review ongoing initiatives within the public healthcare sector in South Africa and their influence to provide future direction.

**Method:** Principally a structured review of current and planned activities.

**Results:** There have been a number of major activities and initiatives surrounding the availability and access to medicines in the public system in recent years in South Africa. This includes a National Surveillance Centre and an innovative early warning system for the supply of medicines as well as the development of a National Health Care Pricing Authority and initiatives to improve contracting. There have also been developments to improve the supply chain including instigating Medicine Procurement Units in the provinces and enhancing forecasting capabilities. Access to medicines is improving though the instigation of stable chronic disease management initiatives to increase the number of external pick-up points for medicines. There are also ongoing programmes to enhance adherence to medicines as well as enhance adherence to the Standard Treatment Guidelines and the Essential Medicines List with their increasing availability. In addition, there is a movement to enhance the role of health technology assessment in future decision making. Hospital initiatives include increased focus on reducing antimicrobial resistance through instigating stewardship programmes as well as improving adverse drug reaction reporting and associated activities.

**Conclusion:** Overall, there are an appreciable number of ongoing activities within the public healthcare system in South Africa attempting to ensure and sustain universal healthcare. It is too early to assess their impact, which will be the subject of future research.

## Introduction

South Africa has extended the share of gross domestic product (GDP) devoted to health over the past two decades. In 1995, 7.42% of GDP was spent on health, whilst currently South Africa spends 8.5–8.9% of its GDP on health (Department of Health, 2015; Jakovljevic et al., 2017), with pharmaceuticals an estimated 10–15% of this. However, there is a marked inequality to medicine access in the country, with private healthcare currently accounting for disproportionate availability of facilities (Table 1). Increasing income disparities amongst citizens, and growing out of pocket expenses for healthcare ($67 per capita in 1995 vs. $80 per capita in 2013 adjusted for purchasing price parity), are challenges to equitable and affordable healthcare for all (Jakovljevic et al., 2017). As the right to health is a constitutional provision in South Africa, there has been a strive towards universal health coverage (UHC) and a unified health system to improve equitable access to medicines to the population as a whole (Department of Health, 2012, 2015; Sekhejane, 2013; Jakovljevic et al., 2017; Perumal-Pillay and Suleman, 2017a,b). Currently, South Africa is considered the flagship national health system in sub-Saharan Africa (Jakovljevic et al., 2017), with its National Department of Health (NDoH) moving towards UHC with the introduction of the National Health Insurance (NHI) initiative (Department of Health, 2015; Perumal-Pillay and Suleman, 2017a). This is likely to continue, certainly in the short to medium term.

**Table 1:** An overview of the South African healthcare system (Jobson, 2015; Department of Health, 2016; Econex, 2016; Jakovljevic et al., 2017; Perumal-Pillay and Suleman, 2017b).

	**South African Healthcare System**	
	**Public Healthcare**	**Private Healthcare**
Funding	Government	Individuals or medical insurance
Hospital capacity	±87,141 beds	±34,600 beds
Population served	82.5–84% (42 million); each public healthcare clinic providing care for on average 13,718 patients	17.5% (8.2 million)
Expenditure per capita on health in 2013 (current US$PPP)	US$543	US$578
Total health expenditure (current US$PPP and million constant 2005US$)	US$28,000	US$31,320
Health information system	Mostly paper-based systems; currently changing	Electronic based system

The current population of South Africa is estimated at 55.91 million, 51% female, predominantly young with 30.1% younger than 15 years and a median age of 25.9 years (Statistics South Africa, 2016). However, after death rates peaked between 2003 and 2005, this is changing with an upward trend in life expectancy, mainly attributable to the marked reduction in mortality from HIV/AIDS with improved treatments and high adherence rates generally in sub-Saharan Africa (Binagwaho and Ratnayake, 2009; Ware et al., 2009; Wang et al., 2016a; NDoH, 2017a).

Sub-Saharan Africa still has the highest burden of infectious disease in the world, with one of four communicable diseases, HIV/AIDS, malaria, lower respiratory infections, or diarrhoeal diseases, currently leading causes of years of life lost due to premature mortality (Lim et al., 2016; Wang et al., 2016a,b; Fullman et al., 2017). Within sub-Saharan Africa, South Africa still has one of the highest disease burdens, with a high prevalence of HIV/AIDS, as well as tuberculosis (TB), resulting in South Africa being 122nd out of 188 countries in terms of the Sustainable Development Goals (SDGs) in 2016 (Wang et al., 2016b; Fullman et al., 2017). It is estimated in South Africa that 7.03 million patients currently have HIV/AIDS, with a prevalence rate of 12.7% in 2016, although this can be as high as 8.4 million (Statistics South Africa, 2016; Wang et al., 2016b). However, improved access to, and uptake of, antiretroviral (ARV) treatment, has enabled HIV positive patients in South Africa to live longer and healthier lives, resulting in a gradual decline in AIDS-related deaths in recent years (Pillay-van Wyk et al., 2016; SAMRC, 2016; Wang et al., 2016a; Kabudula et al., 2017).

There is also a high prevalence of sexually transmitted infections (STIs) among women in South Africa, estimated at 13%, with an incidence rate of 20 per 100 women years (Naidoo et al., 2014; Matsitse et al., 2017). Furthermore, there are increasing concerns with the global growth of antimicrobial resistance (AMR) similarly in South Africa, including multidrug resistant bacteria, fungi, HIV, and TB (Paruk et al., 2012). South Africa has responded through the development of a national AMR strategy (NDoH, 2015a), which is being put into action through the national implementation plan (NDoH, 2015b). This is starting to be addressed through the training of all provincial health care workers on antimicrobial stewardship practices, run by two national training centers (Mendelson and Matsoso, 2015; Sooruth et al., 2015; SAASP, 2017), with regional AMR committees now being established.

There has also been a rise in non-communicable diseases (NCDs) in South Africa in recent years, similar to other BRIC countries as well as global trends (Adebolu and Naidoo, 2014; Jakovljevic and Milovanovic, 2015; Kabudula et al., 2017; NCD Risk Factor Collaboration (NCD-RisC)—Africa Working Group, 2017; Rampamba et al., 2017). Overall, up to 78% of people aged 50 or over have hypertension in South Africa, resulting in hypertension being the seventh cause of death and a major contributor to cardiovascular disease in South Africa (Lloyd-Sherlock et al., 2014; Statistics South Africa, 2015). In addition, up to 70% of women and a third of men are currently overweight or obese in South Africa (Cois and Day, 2015).

Consequently, healthcare in South Africa is faced with major challenges arising from a high burden of disease including HIV/AIDS (Wang et al., 2016b), TB, high maternal, neonatal, and child morbidity, high levels of trauma and violence, and growing rates of NCDs (Adebolu and Naidoo, 2014; Naidoo et al., 2014; Jakovljevic and Milovanovic, 2015; Lalkhen and Mash, 2015; SAMRC, 2016; Fullman et al., 2017). These challenges have a profound effect on the health of the South African population, as the country works toward the goal of universal and equitable access to healthcare for all citizens (Department of Health, 2015). The growing burden of NCDs furthermore necessitates health system reform, moving the focus away from prioritizing acute care toward managing complex chronic diseases at the primary health care (PHC) level (Jakovljevic and Milovanovic, 2015).

Initiatives surrounding the NHI build on existing national medicines policies and the Standard Treatment Guidelines (STGs) and Essential Medicine List (EML), which have been implemented in South Africa since 1996 (Gray et al., 2015; Perumal-Pillay and Suleman, 2016, 2017a,b; Matsitse et al., 2017).

Alongside this, there has been the implementation of the Central Chronic Medicine Dispensing and Distribution (CCMDD) programme in South Africa since 2014 to improve access to chronic medicines and enhance patient experiences given the increasing prevalence of NCDs (Department of Health, 2015; Department of Health KZN, 2016a; Perumal-Pillay and Suleman, 2017a). However, there continues to be concern over the level of education of patients and the subsequent impact on adherence to the medicines provided (WHO, 2013; Adebolu and Naidoo, 2014; Nielsen et al., 2017; Rampamba et al., 2017). It is anticipated that improving adherence rates to treatment regimens should reduce morbidity and mortality from NCDs such as cardiovascular diseases (Krousel-Wood et al., 2004; Calhoun et al., 2008; Nielsen et al., 2017). Patient adherence to treatment regimens can also be enhanced through improving patient knowledge and monitoring their care (Moosa et al., 2016; Nielsen et al., 2017), with ongoing initiatives to address this including the potential use of diaries and smart phone adherence applications, given the increasing use of smart phones in South Africa (Africanews, 2016).

Since 2011, a concerted effort has been made by the South African NDoH to ensure quality healthcare delivery at all levels of care, with the publication of the National Quality Standards for Health, based on seven domains and six priority National Core Standards (NDoH, 2011). The public health domain stipulates how health facilities should function together with local communities and relevant sectors, to promote health, prevent illness and reduce further complications; and ensure that integrated and quality care is provided for their whole community. The patient safety, clinical governance, and clinical care domains provide quality criteria to ensure quality nursing, clinical care and ethical practice; reduce unforeseen harm to healthcare users or patients in identified cases of greater clinical risk; prevent or manage problems or adverse events; and support any affected patients or staff (Sekhejane, 2013).

Quality standards are also provided for infection control, clinical support services and measures to enhance the prescribing of medicines according to treatment guidelines (NDoH, 2011). In the future, all health facilities must be fully compliant with national norms and standards to be accredited for providing services within the NHI. These norms and standards are regularly updated and are now promulgated as regulations to the National Health Amendment Act, 2003 (Act No. 61 of 2003) (NDoH, 2017b).

There is growing evidence that access to high-quality healthcare substantially improves health outcomes for patients including those with infectious diseases, maternal health, cancers and NCDs (Barber et al., 2017). Access and quality are important priorities for UHC, moving towards achieving identified SDGs (Barber et al., 2017; Fullman et al., 2017). Consequently, quantifying the extent to which personal healthcare and access can improve population health, and ultimately health-system performance, is an important priority. However, at this stage, the SDGs' UHC target is focused mainly at tracer interventions in areas of maternal and child health, reproductive health, and a subset of infectious diseases, without adequately recognizing the essential role of personal healthcare in combating NCDs and injuries (Lim et al., 2016; Barber et al., 2017; Fullman et al., 2017). One way forward is to quantify personal healthcare access and quality (Barber et al., 2017), with changes in the Healthcare Access and Quality (HAQ) Index over time indicating where personal HAQ have improved in parallel with changes in development. In the case of South Africa, although there has been a marked improvement in the HAQ Index since 1990, research shows that greater gains in personal HAQ should still be possible given South Africa's place within the development spectrum (Barber et al., 2017).

Ensuring adequate supplies of medicines, at affordable prices, is a key element of managing diseases, including NCDs, effectively. To ensure this, comprehensive procurement systems and robust supply chain management systems are key to manage those medicines deemed essential as per the STGs. Functioning logistic networks will improve access to medicines, and innovative programmes to assess medication usage will lead to better understanding of how rationally medication is being used in a country, as well as ensuring sustainability (Department of Health, 2012, 2015).

South Africa is continually developing and evaluating programmes to monitor the care of patients and to assess the use of medicines in the country, from improving antimicrobial use (Messina et al., 2015, 2017; Brink et al., 2016), to improving the functionality of Pharmaceutical and Therapeutics Committees (PTCs) at all healthcare levels (Gauteng, 2004; Matlala et al., 2017a), and ongoing pharmacovigilance programmes (Suleman, 2010; Mehta et al., 2014; MCC, 2016; Gauteng, 2017).

Consequently, the objective of this paper is to review ongoing initiatives within the public healthcare sector in South Africa in recent years to improve the quality of care, the implications and influence where known, as well as potential future developments. As a result, to provide a platform for debating these issues among key stakeholders in South Africa to help improve future healthcare delivery efficiently, given the paucity of publications assessing pharmaceutical policy in developing countries (Gray and Suleman, 2015).

## Methods

This is principally a structured review of current activities, and their influence and impact where known, rather than an extensive literature search of peer-reviewed publications. We have used similar approaches in previous publications (Dylst et al., 2013; Godman et al., 2013, 2014; Malmstrom et al., 2013) to provide a platform for describing and debating key issues to improve the future quality and efficiency of care, especially regarding medicines.

## Findings

There have been a number of major activities and initiatives surrounding the availability of, and access to, medicines in the public system, as well as initiatives to improve patient care and safety through guidelines, PTCs as well as pharmacovigilance initiatives, in recent years in South Africa. These initiatives are expected to continue as part of attaining and maintaining good quality healthcare for all.

### Availability and supply of medicines

The medicine value chain plays a critical role in the overall performance of any health system. Consequently, there is a need to ensure patients have a dependable supply of the right medicines, available at the right time, in the right quantity, and at the right place.

There are a number of innovations that have already been implemented in South Africa in recent years to improve medicine availability and access. A National Surveillance Centre and innovative early warning system has now been established in South Africa, with dashboards showing medicine stock levels at PHC facilities, hospitals and suppliers throughout the country. This system uses mobile applications, or electronic systems, to gather information and generate warnings where shortages are likely to occur.

**Figure 1:**
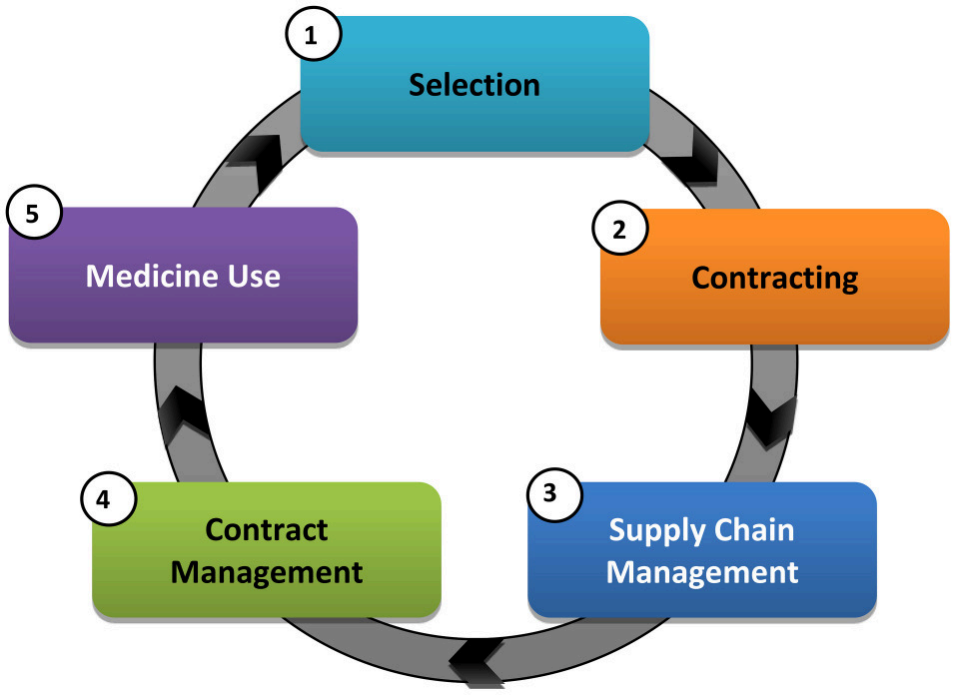
Core medicine value chain functions.

The NDoH in South Africa has also been piloting and implementing a number of health system strengthening reforms, aimed at improving medicine availability and use in recent years that center on five core medicine value chain functions (Figure 1), which can be further sub-divided.

The new strategies aim to achieve a continued dependable supply and reliable payment for goods and services, as well as improved process efficiencies and governance. These changes will assist South Africa in servicing the growing patient needs with the implementation of NHI in a responsive manner. The overall intention is also to improve the scrutiny over current medicine prices and their availability with the development of a National Health Care Pricing Authority, in addition to proposed changes in supply chain and contracting arrangements. As a result, address concerns to access and affordability of medicines, especially essential medicines (Perumal-Pillay and Suleman, 2017a).

#### Interventions to improve contracting

Most essential medicines in South Africa are sourced through a centralized, open tendering process where contracts are entered into between the NDoH and suppliers of medicines, on behalf of all provinces. With the strategic use of market intelligence, improved competition and efficiencies associated with pooled volumes, low prices can be achieved (Pharasi and Miot, 2012).

##### Design and implement differentiated contracting mechanisms

Currently, the majority of pharmaceuticals are contracted through an open tendering processes in South Africa (Pharasi and Miot, 2012). This “one-size-fits-all” approach is, however, not always suitable. The National Treasury's 2015 Supply Chain Management Review Report highlighted the need to implement “strategic sourcing,” allowing a differentiated approach to contracting that considers the strategic importance of the product being purchased, as well as the complexity of the supplier market (Department National Treasury - Republic of South Africa, 2015). To sustain low medicine prices, as well as a reliable supply, the NDoH in South Africa will be implementing an evidence-based, differentiated approach to contracting in consultation with the National Treasury Office of the Chief Procurement Officer (Department National Treasury - Republic of South Africa, 2015).

Revised contracting mechanisms will also be developed based on a current review. It is likely that different approaches to contracting will be established for different medicine “types” to enhance the availability and affordability of essential medicines.

##### Implement targeted international and local price benchmarking to ensure sustained affordability

The use of price benchmarking, similar to other countries (Leopold et al., 2012), as part of a strategic sourcing strategy will assist the NDoH in reviewing price trends, including areas of risk and opportunity. This approach will be adopted as a policy tool to ensure long-term affordability of pharmaceutical services.

#### Interventions for supply chain optimization

Currently, the medicine supply chain in South Africa is characterized by limited planning activities, and outdated duplicative processes, with high variability among the provinces and districts. Additionally, storage infrastructure appears ill-equipped to fully service the growing disease burden and programme requirements of South Africa. This will change in the future with a number of planned developments, which are described below.

##### Expand functionality of Medicine Procurement Units to oversee and manage all supply chain functions

Medicine Procurement Units (MPUs) are the cornerstone of the new supply chain in South Africa, responsible for providing shared planning and operational management services regardless of medicine type, point of use, or methods of replenishment and distribution.

**Figure 2:**
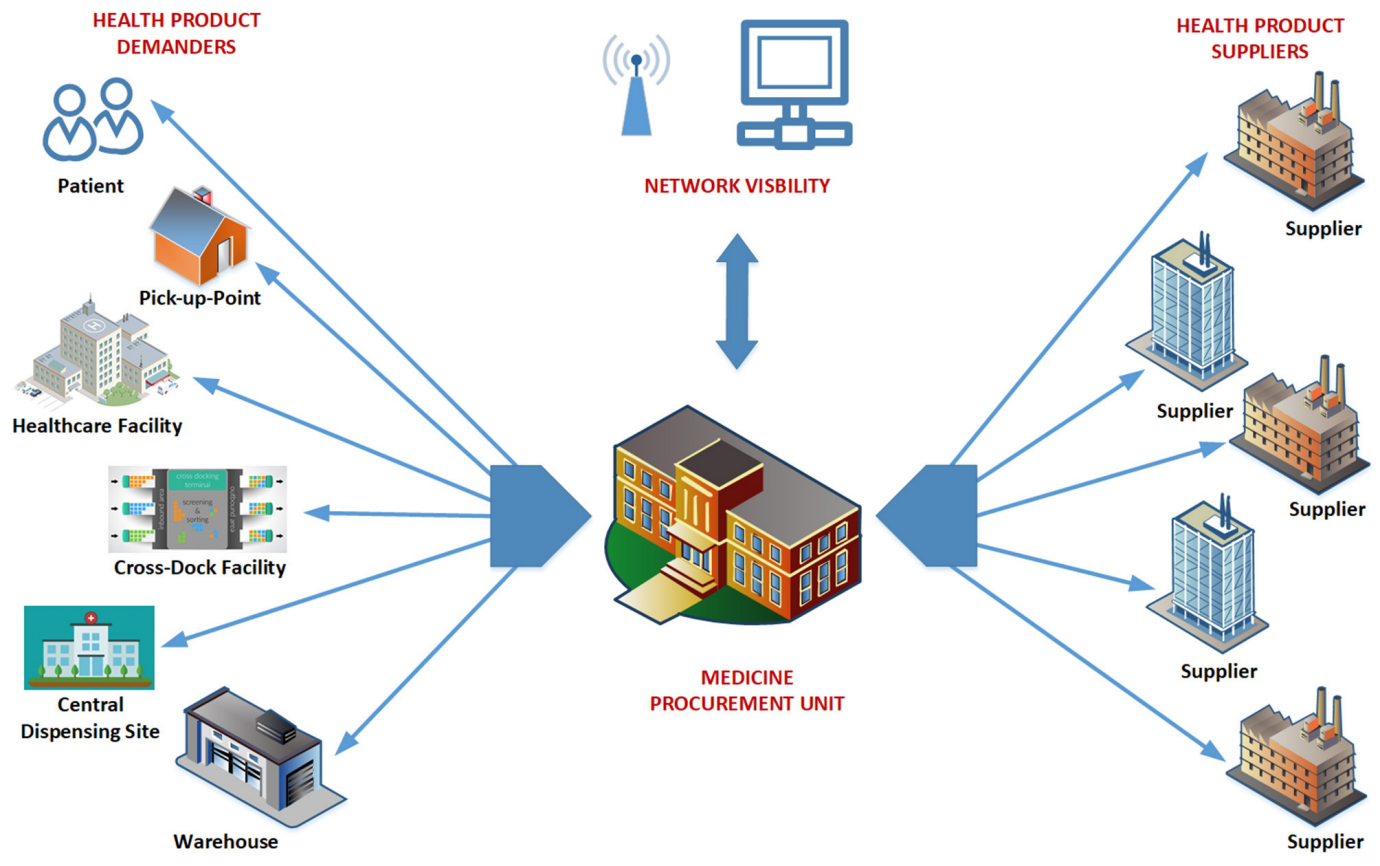
Activities of the Medicine Procurement Unit.

Figure 2 illustrates the visibility of the MPUs. For efficient, co-ordinated services, MPUs will have full control and visibility for their given jurisdiction. Networks of MPUs should ultimately be able to account for all public sector medicine transactions.

The MPUs are responsible for provincial contract management and monitoring of supply chain performance, with MPUs now fully implemented with conditions of contract for every supplier. The accuracy of any forecasting plans will be monitored by the MPUs, and ultimately at a national level, to improve future medicine availability and prices.

To guide differentiated approaches to the implementation of the various reforms and initiatives, a supply chain segmentation approach has been adopted, coordinated through the MPU. These supply chain “segments” will now be managed more efficiently to improve product availability.

##### Implement collaborative Demand Forecasting and Planning processes between provinces

Demand Forecasting and Planning (DFP) are the processes used to estimate patient needs using statistical forecasting techniques. A Demand Forecast enables the government in South Africa to mobilize the investments needed to meet the health needs of the population.

Where medicines have been identified as needed, but have limited or no availability in South Africa, the Demand Forecast signals potential sales to the market, and allows suppliers to plan their investments and business strategies. Collaborative DFP processes will be established among the provinces in South Africa, with the national group overseeing its implementation.

##### Implement supply planning processes

Supply planning initiates responses to identified requirements, outlined in the Demand Plan, and progresses from the current situation where facilities are typically over, or under, stocked. This will be overseen by the relevant MPU (Figure 2).

##### Implement improved inventory management

To adopt effective supply planning, inventory management must be improved. Processes will include reviewing necessary minimum infrastructure requirements for storing medicines appropriately in compliance with legislated norms and standards for health establishments in South Africa (NDoH, 2011, 2017b), and ensuring that all health establishments are adequately resourced to comply with these standards.

##### Implement improved replenishment management

Currently most public sector healthcare facilities in South Africa are replenished through a uninformed “pull replenishment mechanism,” initiated by facility staff equipped with limited supply chain training. To address concerns with unpredictable ordering and stock levels, the MPU will provide a shared planning function for replenishment management, with the aim of implementing informed push, or advised pull, to improve medicine supply.

##### Strengthen distribution planning and differentiated physical distribution channels

Addressing challenges with inappropriate stock levels and wastage is growing in importance in South Africa with the expansion of chronic disease management programmes (Perumal-Pillay and Suleman, 2017a; Rampamba et al., 2017), and the implementation of Test and Treat for HIV/AIDS, that was initiated in September 2016 (Durban, 2016). To address existing challenges, and expand the capacity of the supply chain, three distribution channels have been identified:

- Direct delivery
- Central dispensing for patients
- Warehouse distribution

**Figure 3:**
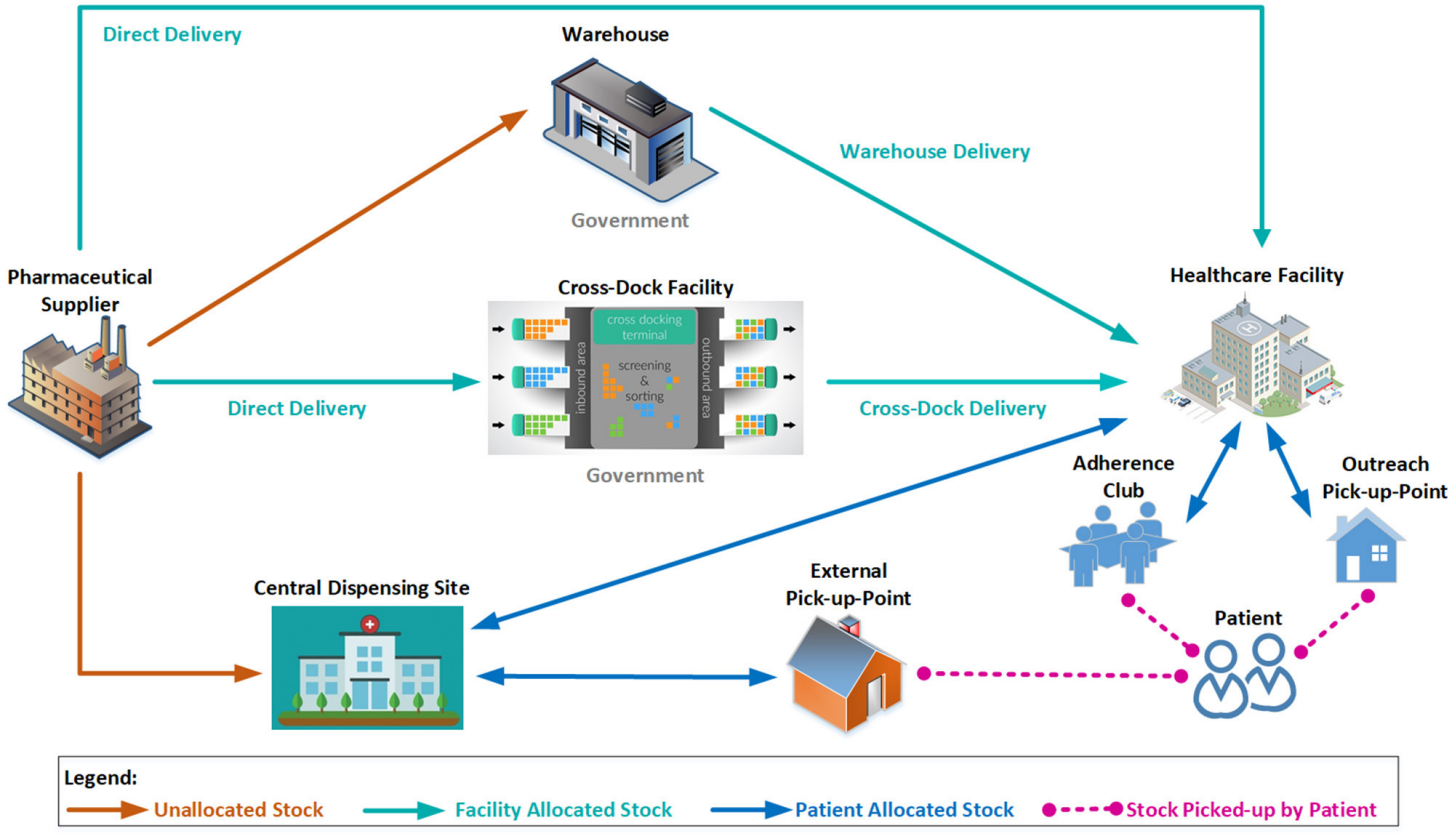
Stylized physical distribution channels.

Distribution planning will be introduced by the MPUs to ensure that distribution processes are optimized across all channels. Figure 3 illustrates some of the possible combinations of available channels, all of which will be overseen by the relevant MPU. Contracted suppliers will be required to retain specified levels of finished products to improve cash flow as well as reduce stock loss and damage. This will result in healthcare facilities having the peace of mind regarding stock being available and delivered within contractual lead times. This will be linked to demand and supply plans as part of an overall contract management function to improve the distribution system.

In order to further improve the supply chain responsiveness, and improve access for patients, central dispensing of chronic medication to an external pick-up point (PuP) convenient for the patient has already been introduced (Department of Health, 2015), freeing healthcare workers to attend to patients who require acute clinical attention (see section Access to Medicines). Due to improved and easier access to medicines, patient adherence to treatment should be improved, which will result in better health outcomes for patients with chronic NCDs.

#### Interventions for improved contract management

National contracts help leverage economies of scale to achieve price efficiencies by pooling the demand of all provinces. Currently, there is fragmentation in contracting due to a lack of standard processes and systems. A National Contract Management Unit (CMU) will monitor information aggregated through MPUs, who will be responsible for implementing tactical, uniform contract management protocols, as a shared service on behalf of the health facilities within their jurisdiction. The shared function between CMU and MPUs is already in place. However, it is recognized that this development will require agile institutional processes, capable of responding to the changing market dynamics to improve future contracting. Processes will be helped by the fact that the information gathered will be used to monitor agreed performance metrics, with pertinent changes implemented to improve future performance if needed.

The relevant MPUs will be responsible for implementing tactical, uniform contract management protocols, as a shared service on behalf of the health facilities within their jurisdiction.

Although, there are many interventions that are already underway, the complexity of the environment means that there is still a long way to go. Addressing root causes of current problems over the short, medium and long term, will require agile institutional processes, capable of responding to the changing market dynamics that are expected to accompany the implementation of NHI. The new strategy being implemented in South Africa aims to achieve a continued dependable supply and reliable payment for goods and services, improved process efficiencies and governance, predictable demand and reduced wastage. These changes will assist South Africa in servicing the growing patient needs, including those for NCDs, with the implementation of NHI in a responsive manner.

The overall intention is to improve the scrutiny over the current prices paid for medicines in South Africa with the development of a National Health Care Pricing Authority (Department National Treasury - Republic of South Africa, 2015) and a National Health Pricing Advisory Committee, which will be a ministerial advisory committee (MAC), within the implementation structures of the NHI (NDoH, 2017c). This is in addition to ensuring continued access to, and supply of, agreed medicines.

#### Surveillance systems for medicines

Surveillance systems for medicines include a visibility and analytics network, which consists of various dashboards including the assessment of medication availability at PHC and hospital level, and data triangulation, as well as prescribing data, guidelines and prices, to improve future medicine procurement and use.

Supplier performance measurement will be derived from the South African Pharma Deliveries Data, with performance scorecards as outputs measuring Lead Time, On Time and In Full (OTIF) deliveries coupled with timely monthly reporting. In addition, a Pipeline Analysis Tool (PAT) has been developed with the data submitted by suppliers on a fortnightly basis. The visibility of supply vs. demand from suppliers are reported with forward production planning for at least three months. An Age Analysis Tool (AAT) has also been developed with the data being supplied by suppliers giving an indication of outstanding debt for contracted items. The data will be triangulated with PMPU stock availability data as well as Hospital and PHC data to add robustness. This will now provide a proactive response in terms of stock outs and low stock holding to improve future supplies.

By July 2016, the surveillance systems covered all 3,400 PHC facilities in South Africa and two thirds of hospitals, with the initial focus on HIV, TB and vaccines. This is currently being expanded to other essential medicine items. A key lesson has been the essential need to establish a master procurement catalog and bar coding system for nation-wide roll out. Barcodes are seen as essential to track medicines through the value chain.

### Access to medicines

**Figure 4:**
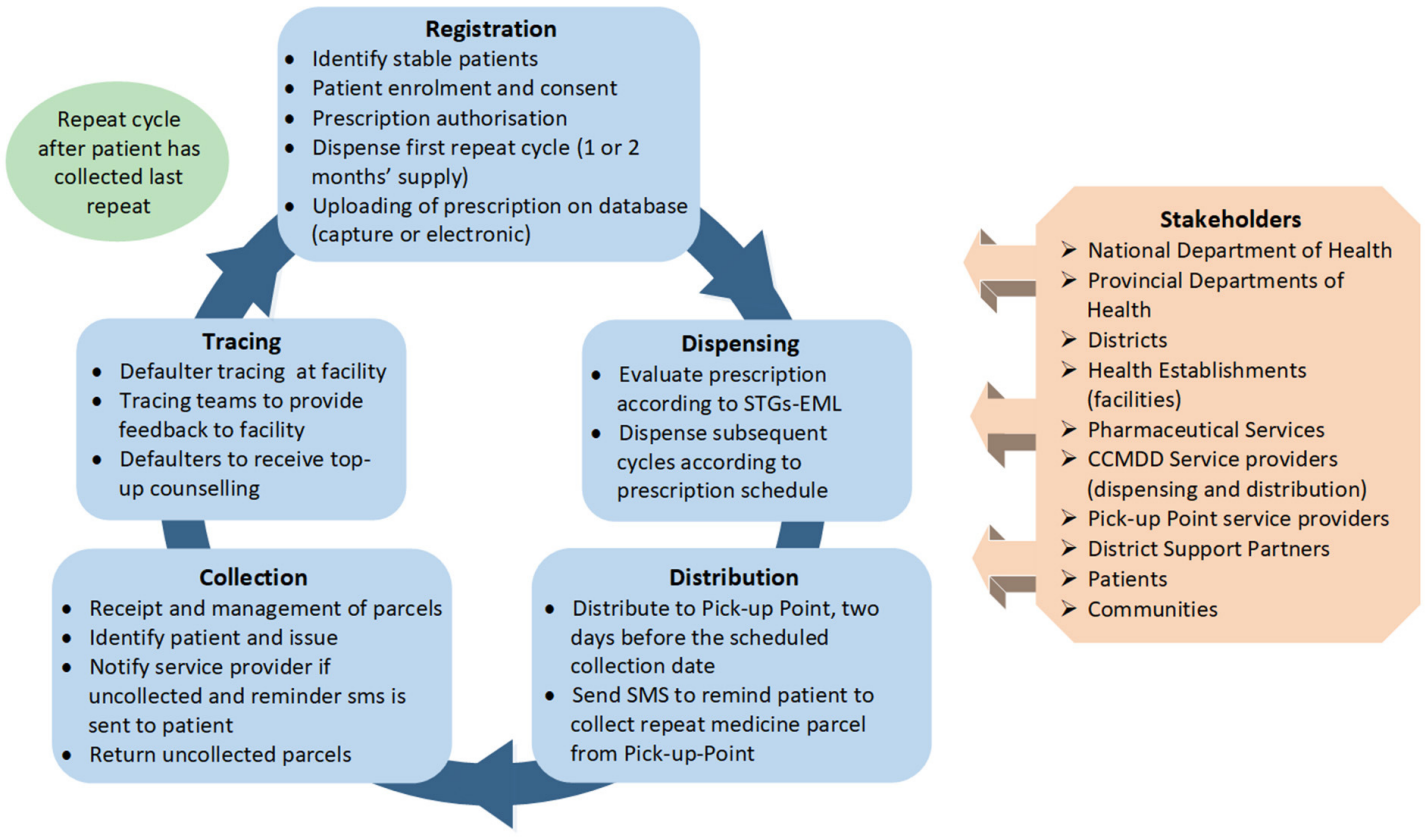
Flow diagram of the Central Chronic Medicines Dispensing and Distribution (CCMDD) process.

The changing epidemiological profile of South Africa has led to an over extension of services delivered by public sector healthcare facilities, with subsequent enormous strain on available resources that have contributed towards medicine shortages and challenges in the quality of care provided. The Central Chronic Medicines Dispensing and Distribution (CCMDD) programme was implemented to enhance access to medicines for stable patients on ARVs, patients on ARVs with comorbidities as well as patients with NCDs requiring chronic therapy (Magadzire et al., 2015; Perumal-Pillay and Suleman, 2017a; Rampamba et al., 2017), with its process flows and content illustrated in Figure 4.

#### Phases and outcome from the implementation of the CCMDD programme

The CCMDD programme was initially implemented in ten NHI districts in February 2014. The Western Cape province was not included due to the fact that they already had implemented a similar programme (Magadzire et al., 2015). Due to the success of the CCMDD programme (Department of Health KZN, 2016b), it has since been rolled out to 46 districts across the other eight provinces. Each province has autonomy regarding the conditions to be managed through the CCMDD programme.

According to service provider reports, more than 1.7 million patients have been registered on the programme in the eight provinces. The NDoH has also appointed more than 650 external PuPs. Additionally, patients can also make use of Adherence Clubs and outreach PuPs to collect repeat medicines, and these collection points are managed by the various facilities (see Figure 3). As a result, access to, and use of, medicines within the public healthcare sector should appreciably improve.

#### Available indicators

Monthly statistics are now available to assist provinces and government in keeping track of the system and patients to improve future care. These include the following indicators:

- Number of patients registered per facility/district/province
- Number of districts registered per province
- Number of facilities registered per district/province
- Number of external PuPs appointed per district/province

Additional indicators include the following:

- Number of parcels delivered per PuP or per district/province
- % of patients on ARVs
- % of patients on ARVs with comorbidities
- % of patients with NCDs
- % of patients collecting from external PuPs

An alternative model for structured, safe, and reliable service delivery closer to a patient's workplace or home, is the Remote Automated Dispensing Units (RADUs) (Toit, 2017). However, only a few of these units are currently being piloted in South Africa, and it is too early to comment. In terms of future service delivery, the aim of this type of model is also to create a controlled method whereby chronic disease patients can, for example, have screening tests performed when they collect their chronic medicine parcels (Toit, 2017). This could be an opportunity for community pharmacies within the private healthcare sector to introduce an affordable model or service package, which could be purchased within the NHI system to improve access to medicines and healthcare (Toit, 2017).

### Initiatives to improve the quality and efficiency of patient care

#### Standard treatment guidelines and essential medicine list

The Standard Treatment Guidelines and Essential Medicine List (STGs-EML) have been developed in South Africa to ensure medicines are available which are safe, effective, and cost-effective, and promote the rational use of medicines (Gray et al., 2015; NDoH, 2015c; Sooruth et al., 2015; Perumal-Pillay and Suleman, 2016, 2017b). STGs-EML have been developed for all levels of healthcare, namely PHC (focusing on nurse-initiated treatment), hospital level guidelines for pediatrics and adults, and a tertiary/quaternary EML for specialist level (Pharasi and Miot, 2012), through their respective Expert Review Committees (ERCs). The ERCs are under the direction of the South African National Essential Medicines List Committee (NEMLC), and consist of key stakeholders relevant for their particular level of care, such as cardiologists for hospital level and nursing prescribers for the PHC level (Leong et al., 2015).

Key principles used in the development of the STGs-EML include evidence-based decision making, with criteria based on efficacy, safety, affordability, cost-effectiveness, and implications for practice. Evidence and final medicine selections are peer reviewed to enhance the robustness of decision making. The STGs-EML are reviewed by the ERCs on a three-yearly basis, with two opportunities in the process for South African healthcare workers to provide comments. Previously published in book format after the three year review process, completed chapters are now released on a mobile phone application, the EML Clinical Guide, allowing health care workers and patients immediate access to the latest medicines and guidelines in the public healthcare sector. This mobile application is now the primary reference source for healthcare workers to access the STGs-EML (Lancaster, 2016) with the increasing use of smart phones in South Africa. This is expected to enhance adherence to current guidelines, which has been variable in practice (Sooruth et al., 2015; Matsitse et al., 2017).

Recommendations for new medicines to be placed on the STGs-EML are received by the ERCs from provincial PTCs within hospitals or from ambulatory care clinics through their district PTCs. A recent comprehensive publication by Perumal-Pillay and Suleman documented that in recent years the number of suggested medicines for PHC facilities within the PHC STGs-EMLs has increased whilst the number has decreased for adult and pediatric EMLs (Perumal-Pillay and Suleman, 2016). Comprehensive interviews with key members of the NEMLC ascertained that the processes have changed over the years to become predominantly evidence-based (Perumal-Pillay and Suleman, 2017b). The quality, safety and efficacy of new medicines for potential inclusion are typically considered first followed by cost considerations (Perumal-Pillay and Suleman, 2017b), although affordability is still a major area for sustainability and access to all.

The development of guidelines with all key stakeholder groups can include criteria for use as well as criteria restricting the use of medicines to defined populations, including potential rationing criteria. The goal of the STGs-EML is enhanced by developing a well thought out and evidence-based list of key medicines, which is peer reviewed, to meet the needs of the population, similar to other countries including Spain, Sweden, and the United Kingdom (Norman et al., 2009; Gustafsson et al., 2011; Bjorkhem-Bergman et al., 2013).

Other healthcare systems have faced similar challenges to South Africa in terms of limited budgets and increased demand for healthcare (Jakovljevic et al., 2016). This has resulted in policies to enhance cost-effective prescribing among Western European countries, including enhancing the use of generics (Godman et al., 2014), to help maintain universal and comprehensive healthcare across populations. This is in contrast to Central and Eastern European (CEE) countries with high co-payments, with more limited public spending (Jakovljevic et al., 2016). Such divisions have resulted in for instance appreciably lower use of biological medicines to treat inflammatory diseases such as rheumatoid arthritis and inflammatory bowel diseases among CEE vs. Western European countries (Putrik et al., 2014a,b; Kostic et al., 2017). The ongoing measures in South Africa, including chronic disease management programmes alongside NHI initiatives, should help address concerns seen among CEE countries.

With the implementation of NHI, the NDoH will be appointing a MAC on Health Technology Assessment (HTA). This multi-disciplinary committee will provide recommendations to the Minister of Health on the prioritization, selection, distribution, management and introduction of interventions for health promotion, disease prevention, diagnosis, treatment, and rehabilitation (NDoH, 2017c). Part of this will include the creation of a South African HTA Agency (NDoH, 2017c). The MAC-HTA will be involved in both investment and disinvestment decisions, similar to agencies in other countries (Parkinson et al., 2015; Guerra-Junior et al., 2017).

#### Integrated systems approach to improve efficiency of access and care

**Figure 5:**
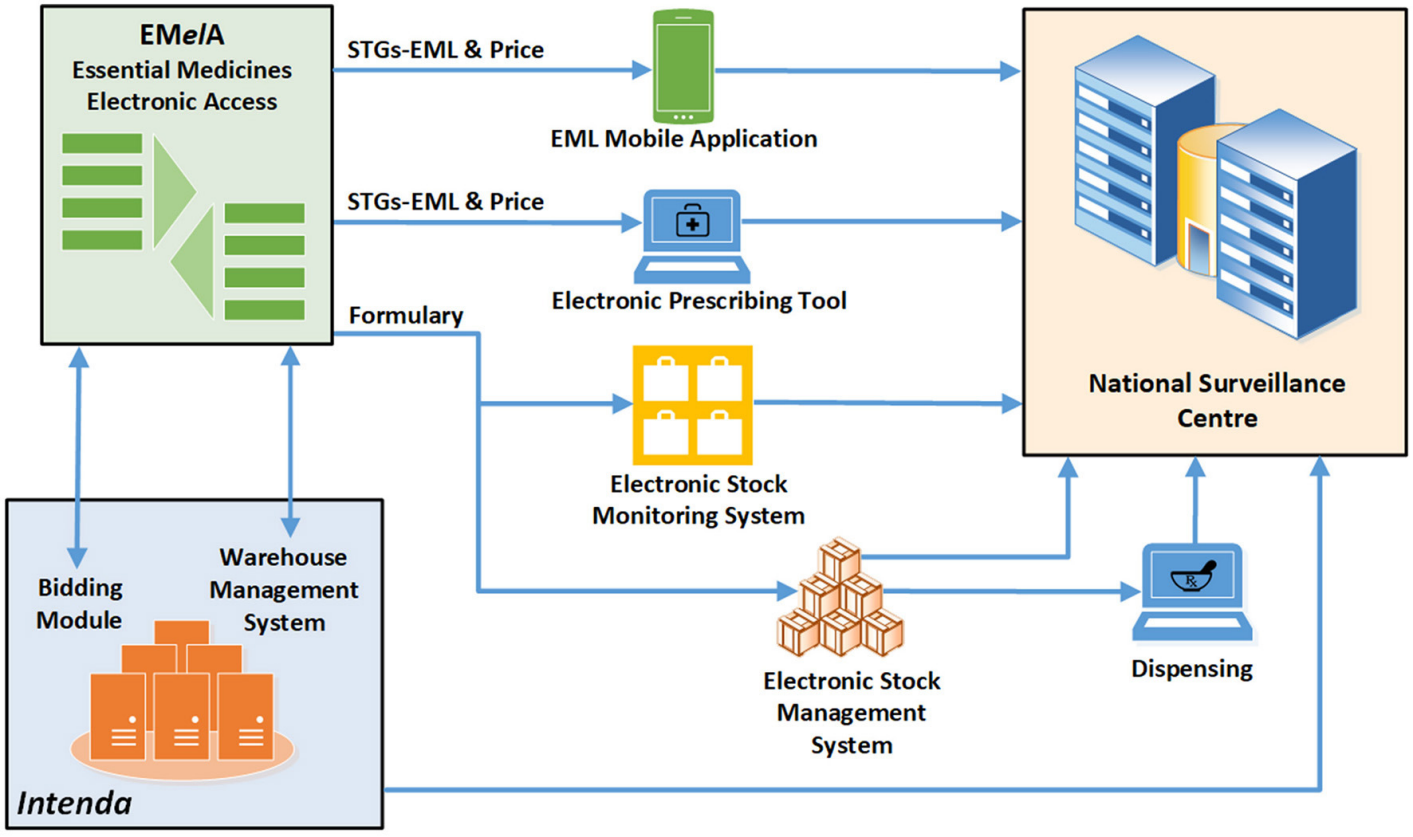
Integrated systems approach to improve efficiency of access to medicines.

Improved access to medicines will in future be strengthened in terms of efficiency by the use of innovative technology to support the selection of medicines, formulary management, standard treatment regimens for prescribers, rational medicine use interventions, and adherence support. An integrated online platform, Essential Medicines Electronic Access (EMelA), currently under development will be integrated with various electronic platforms, including electronic prescribing and dispensing tools, patient management systems and stock management systems (Figure 5).

Alongside these changes, there will be ongoing programmes to enhance compliance to the STGs. This includes possibly educational and other activities including pharmacists and other key stakeholders where there are concerns with prescribing, which is likely to include academic detailing similar to other countries (Costa et al., 2016). Ongoing research programmes will include seeking ways to further improve compliance to prescribing guidance to improve future care where there are identified concerns (Sooruth et al., 2015; Matsitse et al., 2017). Instigation of electronic prescribing and monitoring of prescriptions against agreed guidance will also enhance future adherence to prescribing guidance, as seen for instance with the “Wise List” in Stockholm County Council in Sweden (Gustafsson et al., 2011; Bjorkhem-Bergman et al., 2013; Eriksen et al., 2017).

#### Programmes to improve adherence and retention in care

With growing numbers of patients accessing treatment for HIV, TB, and NCDs, adherence increasingly becomes a challenge for all patients with chronic diseases (Rampamba et al., 2017). Despite South Africa having the largest ARV treatment programme in the world, with generally better adherence rates in sub-Saharan Africa compared with North America (Binagwaho and Ratnayake, 2009), challenges such as declining retention in care rates, and increased drug resistance due to poor adherence among some patients and poor programme performance are evident (Awolola et al., 2014; Baloyi et al., 2014; Ramdas et al., 2015; Vagiri et al., 2015).

To address these concerns, the South African NDoH recently introduced their National Adherence Guidelines for Chronic Diseases including HIV, TB, and NCDs to enhance treatment adherence, working towards Vision 2030 of the National Development Plan (Republic of South Africa, 2012; NDoH, 2016). These guidelines aim to strengthen access to appropriate services in order to improve clinical outcomes, and for people with chronic diseases ensure linkage to care, retention in care, and support, to enhance their adherence to treatment (NDoH, 2016), with enhanced adherence improving outcomes (Ho et al., 2009; Corrao et al., 2011; Abegaz et al., 2017).

Overall, the aim is to introduce a minimum package of interventions to ultimately support linkage to care, adherence and retention in care, to improve future patient outcomes. This includes standardized education counseling on hypertension, diabetes, TB and HIV, including child and adolescent disclosure counseling. In addition, alternative repeat prescription collection strategy options (see section Access to Medicines), early tracing and retention in care as well as integrated management of chronic conditions involving multi-disciplinary groups. This will include patient adherence plans and pamphlets, adherence information, training files, standard operating procedures, fast track initiation counseling, and the setting of treatment goals (Toit, 2017).

#### Antimicrobial stewardship programmes

There are ongoing programmes within hospitals in South Africa to instigate antimicrobial stewardship programmes (ASPs) to reduce AMR (Brink et al., 2016; SAASP, 2017), which is a growing problem worldwide (WHO, 2014, 2015; Hoffman and Outterson, 2015; Mendelson and Matsoso, 2015; NDoH, 2015a,b). Antimicrobial stewardship refers to coordinated interventions designed to improve and monitor the appropriate use of antibiotics by promoting the selection of the optimal antibiotic agent(s), dose, duration of therapy, and route of administration. The large burden of infectious diseases in South Africa has necessitated the following specific antimicrobial stewardship goals: optimizing therapy for individual patients, preventing overuse and misuse of antibiotics, and minimizing the development of resistance at patient and community health levels (NDoH, 2015a,b).

This increasingly includes utilizing existing resources of pharmacists, registered nurses and other healthcare programmes to co-ordinate anti-infective management programmes and subsequently improve patient outcomes (Schellack et al., 2016; Goff and Mendelson, 2017). Major shifts in policy toward combatting AMR within the South African healthcare environment culminated in the NDoH's publication of South Africa's Antimicrobial Resistance National Strategy Framework 2014-24, published in October 2014 at a Ministerial Antimicrobial Resistance Summit to retain the effectiveness of available antibiotics (NDoH, 2015a) This is a multidisciplinary, intersectoral strategy combining human health, animal health, and environmental health sectors, which is implemented through the MAC on AMR. One of the strategic objectives of the framework was to “promote appropriate use of antimicrobials in human and animal health” through ensuring access to safe, effective and affordable antimicrobials, institutionalizing ASPs across sectors, and addressing the use of antimicrobials in animal health and crop production. This Committee is currently developing a series of guidance documents regarding the governance structures necessary at each level of care, the monitoring of antimicrobial use and resistance at the healthcare level, and the appropriate use of antimicrobials at all healthcare levels, in human and animal health (NDoH, 2015a,b).

Parallel to this, and as part of the country's drive towards quality healthcare service delivery, specific antimicrobial stewardship, and infection prevention control norms and standards were finalized and published for public comment in the Government Gazette, with a view to becoming enforceable regulations in the future. The terms of reference of provincial AMR committees have been developed, and these committees are currently in the process of being established.

The need for pharmacist-led ASPs was demonstrated in a recent study with analysis of 47 private hospitals across South Africa revealing that close to one in every 15 prescriptions contained inappropriately prescribed antibiotics, requiring an intervention. The study highlighted the role pharmacists can play to improve future antibiotic use in South Africa as infectious disease-trained specialists are currently limited in number (NDoH, 2015a,b; Brink et al., 2016). Research is also being undertaken in public hospitals to assess the situation in this important sector (Schellack et al., 2017; Bester et al., in press).

Furthermore, the Committee is currently developing a national AMR and antibiotic use national surveillance dashboard, which includes private and public healthcare sector data. The South African Society of Clinical Pharmacy has also developed a guideline to outline the importance, role and purpose of pharmacists in ASPs within South African hospitals to improve the use of antimicrobials. This is in addition to other strategies as part of the South African government's national strategy initiative to reduce AMR (NDoH, 2015a,b).

#### Pharmaceutical and therapeutics committees

It is also recognized that the role and functionality of PTCs needs to improve within the health system, at provincial, district, and institutional levels, in South Africa to enhance appropriate medication procurement and usage decisions to ensure sustainable financial practices that will improve patient care following the instigation of the National Drug Policy in 1996 (NDoH, 1996; Gray et al., 2015). In light of this, the National Policy for the Establishment and Functioning of Pharmaceutical and Therapeutics Committees in South Africa was published in 2015 (NDoH, 2015d). A guidance document with clearer directives on the functioning of the PTC is currently under development. PTC activities typically include dissemination of decisions from the NEMLC meetings as well as any formulary management changes at their respective healthcare institutions.

However, there are concerns with the level of reporting of adverse drug reactions (ADRs) and medication errors, as well as lack of evidence-based decision-making in formulary management (Suleman, 2010; Matlala et al., 2017a,b; Truter et al., 2017). This follows research highlighting the lack of reporting of ADRs within hospitals in South Africa, exacerbated by lack of knowledge of the processes involved (Matlala et al., 2017a; Terblanche et al., 2017a). This is perhaps not surprising with published research highlighting in South Africa that ADRs frequently develop whilst patients are in hospital (Mehta, 2011; Terblanche et al., 2017a). In addition, in a study conducted among four adult medical wards in South Africa, the authors estimated that ADRs contributed to the death of 2.9% of admitted patients (Mouton et al., 2015). The overall mortality rate was 18 per 100 admissions, with 16% of deaths ADR-related (Mouton et al., 2015). These factors have resulted in concerns regarding the current extent of pharmacovigilance activities in South Africa. However, recent studies among South African teaching and secondary hospitals have shown the benefit of pharmacists' interventions making it easier to detect adverse events, and to classify harm to a particular adverse event, to improve reporting rates and subsequent patient care (Müller et al., 2016; Terblanche et al., 2017b).

#### Pharmacovigilance and patient safety

Previously, pharmacovigilance has been seen as a relatively new science in Africa (Ampadu et al., 2016). This has changed with an appreciable number of African countries now part of WHO Programmes for Drug Monitoring including South Africa (Ampadu et al., 2016). Pharmacovigilance (PV) activities in South Africa have been helped by the formation of a national PV Working Group within the Medicines Control Council (MCC) of South Africa dedicated to this (MCC, 2017), providing direction (MCC, 2012). There have also been regional initiatives to further improve PV and the reporting of ADRs (Gauteng, 2017).

However, despite these initiatives, most healthcare professionals in public sector hospitals in South Africa seem unaware of ADR reporting systems, with only a very limited number of professionals receiving training on ADR reporting, with the vast majority indicating the need for such training (Terblanche et al., 2017a). Following the recent introduction of a pharmacist-driven PV system within a public sector hospital in South Africa, the vast majority of healthcare professionals now support the system (97%), with 85% understanding the need for ADR-reporting. This is helped by 69% now knowing there are ADR forms available (up from 15.2%), with 73% now knowing to whom ADR reports should be submitted (up from 19%). Reasons for non-reporting of ADRs also decreased significantly (Terblanche et al., 2017b).

To further increase access to appropriate reporting channels, access to the national ADR reporting forms are now available on the EML Clinical Guide mobile application. Reports submitted on this application are received by the NDoH Pharmacovigilance Centre for Public Health Programmes and forwarded to the MCC. It is hoped this innovation will also increase ADR reporting as the use of smart phones continues to increase in South Africa.

A pharmacist's participation in the management of hospital in-patients on ARV treatment through the provision of pharmaceutical care has also proven to be beneficial for the identification, management and reporting of ADRs (Ally et al., 2015). This kind of research, and the implications for future activities to reduce the extent of ADRs, and their implications on patient morbidity, mortality and costs, will also be undertaken among public hospitals in South Africa to improve future patient care.

## Discussion and conclusions

There are an appreciable number of ongoing activities within the public healthcare system in South Africa to ensure equitable access to medicines and to improve the care of patients. This follows the instigation of universal access for all patients in South Africa, along with recognition of the growing burden of NCDs in South Africa. Appreciable strides have already been made in reducing the costs of medicines used in the public healthcare sector, particularly ARV treatment for HIV infection.

South Africa has already made some progress towards the SDGs as is evident from an analysis of the health-related SDG indicators based on the Global Burden of Disease studies (Lim et al., 2016; Fullman et al., 2017). Although South Africa's health-related SDG index was below the median for 188 countries (46 vs. 59.3 in 2015 and 122nd out of 188 countries in 2016) (Lim et al., 2016; Fullman et al., 2017), progress has been seen for indicators such as the UHC tracer with a SDG index of 79, similar to the multi-country median. However, still below the UHC tracer target of 100% by 2030 (Lim et al., 2016). Low performance for indicators such as NCDs, childhood obesity, harmful alcohol use, and mortality due to self-harm and interpersonal violence, suggest further interventions are still needed to improve the care of patients in the public healthcare system (Lim et al., 2016; Fullman et al., 2017).

In terms of future estimation of the SDG indicators though, as well as evaluating the influence of future interventions, one should recognize the data gaps with these indicators (Lim et al., 2016). For the UHC tracer indicator, a set of tracer interventions are restricted to reproductive, maternal and child health, as well as a subset of infectious diseases. Furthermore, data on the coverage of NCD interventions and high-level care are limited, as the UHC tracer indicator only captures the interventions used and not the quality of the interventions provided. Future revisions are subsequently required as additional data becomes available on the delivery of interventions to improve the management of NCDs in South Africa, including the chronic disease management programmes as well as the modification of risk factors, with improved management of NCDs a key priority area in South Africa.

Ongoing programmes to improve care in South Africa include the management of chronic diseases incorporating the STGs-EML along with programmes to make access to medicines easier for patients such as the CCMDD programme. In addition, programmes and activities to improve the supply chain as well as continue to obtain essential medicines at low prices. The instigation of central procurement practices ensures the department leverages economies of scale to help to a large extent to procure medicines at optimum prices. There are also ongoing programmes within public hospitals in South Africa to improve the quality of care as well as reduce current AMR and ADR rates. Reducing AMR rates is critical with increasing use of antibiotics, with utilization increasing globally by 36% between 2000 and 2010, with countries such as China, India, and South Africa accounting for 76% of this increase (O'Neill, 2015). Increasing AMR rates pose a serious public health threat as well as increasing costs (Gandra et al., 2014; Hoffman and Outterson, 2015; O'Neill, 2015). In the South African context, especially in the public sector, it is difficult to replicate resource-abundant ASP models. Sustainable and effective ASPs largely depend on monitoring practices, teamwork in developing and implementing interventions, and organizational infrastructure. Consequently, it is critical in the South African context to utilize existing resources such as pharmacists, registered nurses, and other members of the healthcare team, to drive forward ASPs, which is already happening (SAASP, 2017). For effective ASPs in South Africa, it is important to recognize the skills within multidisciplinary teams that can support pharmacists to improve antibiotic prescribing practices within the South African Antimicrobial Resistance National Strategy Framework and its implementation (NDoH, 2015a,b). These are considerations for the future. Potential research activities in the community include a better understanding of key stakeholder attitudes to AMR, and how to develop programmes relevant to their needs, to improve future antibiotic prescribing and dispensing.

HTA will also play an increasingly important role in decision making in South Africa in the future to help assess which future technologies to invest in and which technologies to disinvest in. This will be closely monitored.

Future monitoring of UHC progress in South Africa should combine the HAQ Index, coverage of health interventions, and prevalence of risk factors that can be modified through public health initiatives (Barber et al., 2017). This will provide a more comprehensive measure of the capacity of South Africa's healthcare system capacity to ensure personal HAQ.

Technology will also play a more prominent and important role in the future in South Africa to improve access and availability of medicines, and reduce wastage. As mentioned, a National Surveillance Centre and innovative early warning system has been established, with dashboards showing medicine stock levels at various levels of care throughout the country. Mobile applications or electronic systems are being used to collect information and to alert of possible medicine shortages. Innovative technology is also being used to empower health care workers to provide quality services. The STGs-EML are available as a mobile application, which also allows reporting of ADRs, reporting of stock-outs and provision of information about healthcare facilities. There are also ongoing programmes to improve the management of patients with NCDs, including their education and monitoring of adherence to medicines (Rampamba et al., 2017). In addition, the NDoH will continue to interrogate innovative technologies to improve service delivery and the quality of care. This potentially includes how mobile applications can be used to enhance medicine adherence especially in environments with limited health literacy. Other research programmes include a better understanding of facilitators and barriers to medicine adherence. Consideration of the role of social capital also needs to be better understood in South Africa building on previous research (Binagwaho and Ratnayake, 2009; Ware et al., 2009).

To date, there have been limited formal evaluations of the impact of the extensive number of recent initiatives in South Africa. This is changing, and will be part of future research projects to further improve the quality and efficiency of prescribing in South Africa as the country strives to develop and maintain UHC in the face of an appreciable burden of both infectious and NCDs. Social determinants of health, and the causes of ill health, also need to be investigated further in South Africa with factors such as the level of education and medicine use knowledge substantially impacting on lifestyles and adherence to medicines (Moosa et al., 2015; Rampamba et al., 2017).

## Author contributions

All authors listed have made a substantial, direct and intellectual contribution to the work, and approved it for publication.

### Conflict of interest statement

The authors declare that the research was conducted in the absence of any commercial or financial relationships that could be construed as a potential conflict of interest.

## References

[B1] Abegaz T. M. Shehab A. Gebreyohannes E. A. Bhagavathula A. S. Elnour A. A. (2017). Nonadherence to antihypertensive drugs: a systematic review and meta-analysis. Medicine 96:e5641. 10.1097/MD.0000000000005641 28121920 PMC5287944

[B2] Adebolu F. A. Naidoo M. (2014). Blood pressure control amongst patients living with hypertension presenting to an urban district hospital outpatient clinic in Kwazulu-Natal. Afr. J. Primary Health Care Fam. Med. 6:572. 10.4102/phcfm.v6i1.572 26245402 PMC4502887

[B3] Africanews (2016). South Africa Leads adult Smartphone Use on the Continent. Available online at: http://www.africanews.com/2016/06/01/south-africa-leads-adult-smartphone-use-on-the-continent/

[B4] Ally S. Meyer J. C. Schellack N. Summers R. (2015). Improving patient safety to antiretroviral treatment by actively soliciting adverse drug reaction reports. Afr. J. Phys. Health Educ. Recreation Dance Suppl. 2, 411–428.

[B5] Ampadu H. H. Hoekman J. de Bruin M. L. Pal S. N. Olsson S. Sartori D.. (2016). Adverse drug reaction reporting in Africa and a comparison of individual case safety report characteristics between Africa and the rest of the world: analyses of spontaneous reports in VigiBase®. Drug Saf. 39, 335–345. 10.1007/s40264-015-0387-4 26754924 PMC4796322

[B6] Awolola T. A. Meyer J. C. Summers B. Johnson S. (2014). Identification of over-compliant patients and pill dumpers with the ‘mixed’ pill count method and the impact of counselling or feedback on adherence to antiretroviral treatment. Afr. J. Phys. Health Educ. Recreation Dance. Suppl. 1, 105–115.

[B7] Baloyi G. R. Meyer J. C. Johnson S. (2014). Loss to initiation on antiretroviral treatment (ART) after voluntary counselling and testing (VCT) at two VCT centres near Pretoria, South Africa. Afr. J. Phys. Health Educ. Recreation Dance Suppl. 1, 1–10.

[B8] Barber R. M. Fullman N. Sorensen R. Bollyky T. McKee M. Nolte E. (2017). Healthcare Access and Quality Index based on mortality from causes amenable to personal health care in 195 countries and territories, 1990-2015: a novel analysis from the Global Burden of Disease Study 2015. Lancet 390, 231–266. 10.1016/S0140-6736(17)30818-8 28528753 PMC5528124

[B9] Bester T. Schellack N. Gous A. G. S. Stolz A. Meyer J. C. (in press). Evaluating initial antimicrobial use in an adult intensive care unit at an Academic Teaching Hospital in Pretoria, South Africa. Afr. J. Phys. Act. Health Sci.

[B10] Binagwaho A. Ratnayake N. (2009). The role of social capital in successful adherence to antiretroviral therapy in Africa. PLoS Med. 6:e18. 10.1371/journal.pmed.1000018 19175286 PMC2631052

[B11] Bjorkhem-Bergman L. Andersen-Karlsson E. Laing R. Diogene E. Melien O. Jirlow M.. (2013). Interface management of pharmacotherapy. Joint hospital and primary care drug recommendations. Eur. J. Clin. Pharmacol. 69(Suppl. 1), 73–78. 10.1007/s00228-013-1497-5 23640191

[B12] Brink A. J. Messina A. P. Feldman C. Richards G. A. Becker P. J. Goff D. A.. (2016). Antimicrobial stewardship across 47 South African hospitals: an implementation study. Lancet Infect. Dis. 16, 1017–1025. 10.1016/S1473-3099(16)30012-3 27312577

[B13] Calhoun D. A. Jones D. Textor S. Goff D. C. Murphy T. P. Toto R. D.. (2008). Resistant hypertension: diagnosis, evaluation, and treatment. A scientific statement from the American Heart Association Professional Education Committee of the Council for High Blood Pressure Research. Hypertension 51, 1403–1419. 10.1161/HYPERTENSIONAHA.108.189141 18391085

[B14] Cois A. Day C. (2015). Obesity trends and risk factors in the South African adult population. BMC obesity. 2:42. 10.1186/s40608-015-0072-2 26617987 PMC4603579

[B15] Corrao G. Parodi A. Nicotra F. Zambon A. Merlino L. Cesana G.. (2011). Better compliance to antihypertensive medications reduces cardiovascular risk. J. Hypertens. 29, 610–618. 10.1097/HJH.0b013e328342ca97 21157368

[B16] Costa J. O. Almeida-Brasi C. Godman B. Fischer M. A. Dartnell J. Heaney A. (2016). Implementation of clinical guidelines in Brazil: should academic detailing be used? J. Pharm. Health Serv. Res. 7, 105–115. 10.1111/jphs.12133

[B17] Department of Health (2012). Department of Health Republic of South Africa. National Health Insurance - Healthcare for all South Africans Available online at: http://docplayer.net/1525312-National-health-insurance-healthcare-for-all-south-africans.html

[B18] Department of Health (2015). Department of Health Republic of South Africa. National Health Insurance For South Africa Towards Universal Health Coverage. Available online at: https://www.health-e.org.za/wp-content/uploads/2015/12/National-Health-Insurance-for-South-Africa-White-Paper.pdf

[B19] Department of Health KZN (2016a). Province of KwaZulu-Natal. Launch of the Central Chronic Medication Dispensing and Distribution Programme. Available online at: http://www.kznhealth.gov.za/mediarelease/2016/Launch-CCMDD-10062016.htm

[B20] Department National Treasury - Republic of South Africa (2015). Public Sector Supply Chain Management Review. Available online at: http://www.treasury.gov.za/publications/other/SCMR%20REPORT%202015.pdf

[B21] Department of Health (2016). National Department of Healh, Republic of South Africa. Annexure C: Governance of Public Sector Hospital under the National Health Insurance. Available online at: https://www.google.com/search?client=safari&rls=en&q=NDoH,+2015-Annexure+C_Policy+on+Governance+of+Hospitals_December+2015&ie=UTF-8&oe=UTF-8#q=NDoH,+2015-Annexure+C+Policy+on+Governance+of+Hospitals+December+2015

[B22] Department of Health KZN (2016b). Huge Applause for the KZN Department of Health as Patients Collect Chronic Medication Closer to Home and Save on Traveling Costs. Available online at: http://www.kznhealth.gov.za/mediarelease/2016/Huge-applause-for-the-kzn-department-of-health-a-%20patients.htm

[B23] Durban (2016). SA Adopts “Test and Treat” to Combat AIDS. Available online at: http://architecture.durban.gov.za/Resource_Centre/new2/Pages/SA-adopts-%E2%80%9Ctest-and-treat%E2%80%9D-to-combat-AIDS.aspx

[B24] Dylst P. Vulto A. Godman B. Simoens S. (2013). Generic medicines: solutions for a sustainable drug market? Appl. Health Econ. Health Policy 11, 437–443. 10.1007/s40258-013-0043-z 23846572

[B25] Econex (2016). Competition and Applied Economics. Comments on select aspects of the NHI White Paper. Report on behalf of the Hospital Association of South Africa (HASA) Available online at: https://www.hasa.co.za/wp-content/uploads/2017/06/Econex-NHI-White-Paper-comments-for-HASA_27.05.2016.pdf

[B26] Eriksen J. Gustafsson L. L. Ateva K. Bastholm-Rahmner P. Ovesjo M. L. Jirlow M.. (2017). High adherence to the ‘Wise List’ treatment recommendations in Stockholm: a 15-year retrospective review of a multifaceted approach promoting rational use of medicines. BMJ Open 7:e014345. 10.1136/bmjopen-2016-014345 28465306 PMC5775463

[B27] Fullman N. Barber R. M. Abajobir A. A. Abate K. H. Abbafati C. Abba K. M. (2017). Measuring progress and projecting attainment on the basis of past trends of the health-related Sustainable Development Goals in 188 countries: an analysis from the Global Burden of Disease Study 2016. Lancet 390, 1423–1459. 10.1016/S0140-6736(17)32336-X 28916366 PMC5603800

[B28] Gandra S. Barter D. M. Laxminarayan R. (2014). Economic burden of antibiotic resistance: how much do we really know? Clin. Microbiol. Infect. 20, 973–980. 10.1111/1469-0691.12798 25273968

[B29] Gauteng (2004). Gauteng Province Department of Health. Implementation of Pharmacy and Therapeutics Committees at all Levels. Circular Letter 27. Available online at: http://www.treasury.gov.za/publications/annual%20reports/provincial/2005/GT/GP%20-%20Vote%2004%20-%20Health.pdf

[B30] Gauteng (2017). Gauteng Pharmacovigilance Bulletin. Available online at: file:///C:/Users/mail/Downloads/PV%20Bulletin%2020%20April.pdf

[B31] Godman B. Finlayson A. E. Cheema P. K. Zebedin-Brandl E. Gutierrez-Ibarluzea I. Jones J.. (2013). Personalizing health care: feasibility and future implications. BMC Med. 11:179. 10.1186/1741-7015-11-179 23941275 PMC3750765

[B32] Godman B. Wettermark B. van Woerkom M. Fraeyman J. Alvarez-Madrazo S. Berg C.. (2014). Multiple policies to enhance prescribing efficiency for established medicines in Europe with a particular focus on demand-side measures: findings and future implications. Front. Pharmacol. 5:106. 10.3389/fphar.2014.00106 24987370 PMC4060455

[B33] Goff D. A. Mendelson M. (2017). Antibiotic stewardship hits a home run for patients. Lancet Infect. Dis. 17, 892–893. 10.1016/S1473-3099(17)30344-4 28629875

[B34] Gray A. L. Suleman F. (2015). The relevance of systematic reviews on pharmaceutical policy to low- and middle-income countries. Int. J. Clin. Pharm. 37, 717–725. 10.1007/s11096-015-0156-6 26177819

[B35] Gray A. Suleman F. Patel A. Bannenberg W. (2015). Improving Health Systems Efficiency - South Africa. Implementation of reforms under the National Drug Policy Available online at: http://apps.who.int/iris/bitstream/10665/186477/1/WHO_HIS_HGF_CaseStudy_15.9_eng.pdf

[B36] Guerra-Junior A. A. Pires de Lemos L. L. Godman B. Bennie M. Osorio-de-Castro C. G. S. Alvares J.. (2017). Health technology performance assessment: real-world evidence for public healthcare sustainability. Int. J. Technol. Assess. Health Care 33, 279–287. 10.1017/S0266462317000423 28641588

[B37] Gustafsson L. L. Wettermark B. Godman B. Andersen-Karlsson E. Bergman U. Hasselstrom J.. (2011). The ‘wise list’- a comprehensive concept to select, communicate and achieve adherence to recommendations of essential drugs in ambulatory care in Stockholm. Basic Clin. Pharm. Toxicol. 108, 224–233. 10.1111/j.1742-7843.2011.00682.x 21414143

[B38] Ho P. M. Bryson C. L. Rumsfeld J. S. (2009). Medication adherence: its importance in cardiovascular outcomes. Circulation 119, 3028–3035. 10.1161/CIRCULATIONAHA.108.768986 19528344

[B39] Hoffman S. J. Outterson K. (2015). INTRODUCTION: what will it take to address the global threat of antibiotic resistance? J. Law Med. Ethics 43(Suppl. 3), 6–11. 10.1111/jlme.12267 26243236

[B40] Jakovljevic M. B. Milovanovic O. (2015). Growing burden of non-communicable diseases in the emerging health markets: the case of BRICS. Front. Public Health 3:65. 10.3389/fpubh.2015.00065 25954740 PMC4407477

[B41] Jakovljevic M. Lazarevic M. Milovanovic O. Kanjevac T. (2016). The New and Old Europe: east-west split in pharmaceutical spending. Front. Pharmacol. 7:18. 10.3389/fphar.2016.00018 26973521 PMC4771948

[B42] Jakovljevic M. Potapchik E. Popovich L. Barik D. Getzen T. E. (2017). Evolving health expenditure landscape of the BRICS nations and projections to 2025. Health Econ. 26, 844–852. 10.1002/hec.3406 27683202

[B43] Jobson M. (2015). Structure of the Health System in South Africa. Available online at: http://www.khulumani.net/

[B44] Kabudula C. W. Houle B. Collinson M. A. Kahn K. Gómez-Olivé F. X. Clark S. J.. (2017). Progression of the epidemiological transition in a rural South African setting: findings from population surveillance in Agincourt, 1993–2013. BMC Public Health 17:424. 10.1186/s12889-017-4312-x 28486934 PMC5424387

[B45] Kostic M. Djakovic L. Sujic R. Godman B. Jankovic S. M. (2017). Inflammatory Bowel Diseases (Crohn s Disease and Ulcerative Colitis): cost of treatment in serbia and the implications. Appl. Health Econ. Health Policy 15, 85–93. 10.1007/s40258-016-0272-z 27587010 PMC5253143

[B46] Krousel-Wood M. Thomas S. Muntner P. Morisky D. (2004). Medication adherence: a key factor in achieving blood pressure control and good clinical outcomes in hypertensive patients. Curr. Opin. Cardiol. 19, 357–362. 10.1097/01.hco.0000126978.03828.9e 15218396

[B47] Lalkhen H. Mash R. (2015). Multimorbidity in non-communicable diseases in South African primary healthcare. S. Afr. Med. J. 105, 134–138. 10.7196/SAMJ.8696 26242533

[B48] Lancaster R. (2016). Improving access to health treatment guidelines through mobile technology. S. Afr. Pharm. J. 83, 42–44.

[B49] Leong T. D. Munsamy J. Reddy M. Gray A. (2015). Selection of essential medicines in South Africa – a work in progress. S. Afr. Pharm. J. 82, 39–41.

[B50] Leopold C. Vogler S. Mantel-Teeuwisse A. K. de Joncheere K. Leufkens H. G. Laing R. (2012). Differences in external price referencing in Europe: a descriptive overview. Health Policy 104, 50–60. 10.1016/j.healthpol.2011.09.008 22014843

[B51] Lim S. S. Allen K. Bhutta Z. A. Dandona L. Forouzanfar M. H. Fullman N. (2016). Measuring the health-related Sustainable Development Goals in 188 countries: a baseline analysis from the Global Burden of Disease Study 2015. Lancet 388, 1813–1850. 10.1016/S0140-6736(16)31467-2 27665228 PMC5055583

[B52] Lloyd-Sherlock P. Beard J. Minicuci N. Ebrahim S. Chatterji S. (2014). Hypertension among older adults in low- and middle-income countries: prevalence, awareness and control. Int. J. Epidemiol. 43, 116–128. 10.1093/ije/dyt215 24505082 PMC3937973

[B53] Magadzire B. P. Marchal B. Ward K. (2015). Improving access to medicines through centralised dispensing in the public sector: a case study of the Chronic Dispensing Unit in the Western Cape Province, South Africa. BMC Health Serv. Res. 15:513. 10.1186/s12913-015-1164-x 26577831 PMC4650275

[B54] Malmstrom R. E. Godman B. B. Diogene E. Baumgartel C. Bennie M. Bishop I.. (2013). Dabigatran - a case history demonstrating the need for comprehensive approaches to optimize the use of new drugs. Front. Pharmacol. 4:39. 10.3389/fphar.2013.00039 23717279 PMC3653065

[B55] Matlala M. Engler D. Mashaba T. Maake L. Shihambi E. Netshuingani T. (2017a). Knowledge and Attitudes among Healthcare Professionals to the Reporting of Adverse Drug Reactions at an Academic Hospital, South Africa. MURIA 3; 26 Available online at: http://muria.mandela.ac.za/muria/media/Store/documents/Abstract%20book%20-%20MURAI%203/MURIA3-AbstractBook-July-2017.pdf

[B56] Matlala M. Gous A. G. S. Godman B. Meyer J. C. (2017b). Structure and activities of pharmacy and therapeutics committees among public hospitals in South Africa; findings and implications. Expert Rev. Clin. Pharmacol. 10, 1273–1280. 10.1080/17512433.2017.1364625 28776442

[B57] Matsitse T. B. Helberg E. Meyer J. C. Godman B. Massele A. Schellack N. (2017). Compliance to the primary health care treatment guidelines and the essential medicines list in the management of sexually transmitted infections in correctional centres in South Africa: findings and implications. Expert Rev. Anti Infect. Ther. 5, 963–972. 10.1080/14787210.2017.1382354 28922959

[B58] MCC (2012). Medicines Control Council and National Department of Health. Reporting Adverse Drug Reactions in South Africa. Available online at: http://docplayer.net/37893933-Medicines-control-council.html

[B59] MCC (2016). POST-MARKETING REPORTING OF ADVERSE DRUG REACTIONS TO HUMAN MEDICINES IN SOUTH AFRICA. Medicines Control Council. Available online at: http://www.mccza.com/documents/f39361792.33_ADR_reporting_post-marketing_Jul16_v4.1_showing_changes.pdf

[B60] MCC (2017). Medicines Control Council South Africa. Available online at: http://www.mccza.com/about/

[B61] Mehta U. C. (2011). Pharmacovigilance: the devastating consequences of not thinking about adverse drug reactions. The burden of adverse drug reactions (ADRs) on patient care has been found to be high globally and is particularly high in South Africa. CME 29, 247–251.

[B62] Mehta U. Dheda M. Steel G. Blockman M. Ntilivamunda A. Maartens G.. (2014). Strengthening pharmacovigilance in South Africa. S. Afr. Med. J. 104, 104–106. 10.7196/samj.7517 24893535

[B63] Mendelson M. Matsoso M. (2015). The South African Antimicrobial Resistance Strategy Framework. AMR Control, 54–61.

[B64] Messina A. P. Van Vuuren S. Brink A. J. (2017). Our collective contribution matters: Pharmacists unite in tackling antibiotic resistance for South Africa. S. Afr. Pharm. J. 84, 53–55.

[B65] Messina A. P. van den Bergh D. Goff D. A. (2015). antimicrobial stewardship with pharmacist intervention improves timeliness of antimicrobials across Thirty-three Hospitals in South Africa. Infect. Dis. Ther. 4(Suppl. 1), 5–14. 10.1007/s40121-015-0082-x 26362291 PMC4569642

[B66] Moosa A. Bezuidenhout S. Meyer J. C. (2015). Knowledge of type-2 diabetes among patients attending a Community Health Centre in Pretoria, South Africa. Afr. J. Phys. Health Educ. Recreation Dance Suppl. 2, 241–251.

[B67] Moosa M. S. Kuttschreuter L. S. Rayner B. L. (2016). Evaluation and management of patients referred to a tertiary-level hypertension clinic in Cape Town, South Africa. S. Afr. Med. J. 106, 797–800. 10.7196/SAMJ.2016.v106i8.9610 27499407

[B68] Mouton J. P. Mehta U. Parrish A. G. Wilson D. P. K. Stewart A. Njuguna C. W.. (2015). Mortality from adverse drug reactions in adult medical inpatients at four hospitals in South Africa: a cross-sectional survey. Brit. J. Clin. Pharmacol. 80, 818–826. 10.1111/bcp.12567 25475751 PMC4594724

[B69] Müller M. M. Gous A. Schellack N. (2016). Measuring adverse events using a trigger tool in a paper based patient information system at a teaching hospital in South Africa. Eur. J. Clin. Pharm. 18, 103–112.

[B70] Naidoo S. Wand H. Abbai N. S. Ramjee G. (2014). High prevalence and incidence of sexually transmitted infections among women living in Kwazulu-Natal, South Africa. AIDS Res. Ther. 11:31. 10.1186/1742-6405-11-31 25243015 PMC4168991

[B71] NCD Risk Factor Collaboration (NCD-RisC)—Africa Working Group (2017). Trends in obesity and diabetes across Africa from 1980 to 2014: an analysis of pooled population-based studies. Int. J. Epidemiol. [Epub ahead of print]. 10.1093/ije/dyx078 PMC5837192 28582528

[B72] NDoH (1996). National Drug Policy for South Africa. Available online at: http://apps.who.int/medicinedocs/documents/s17744en/s17744en.pdf

[B73] NDoH (2011). National Core Standards for Health Establishments in South Africa. National Department of Health 2011 Available online at: http://www.rhap.org.za/wp-content/uploads/2014/05/National-Core-Standards-2011-1.pdf

[B74] NDoH (2015a). National Department of Health - Republic of South Africa. Antimicrobial Resistance: National Strategy Framework 2014 – 2024. Available online at: https://www.health-e.org.za/wp-content/uploads/2015/09/Antimicrobial-Resistance-National-Strategy-Framework-2014-2024.pdf

[B75] NDoH (2015b). National Department of Health - Republic of South Africa. Implementation Plan for Antimicrobial Resistance National Strategy Framework 2014-2019. Available online at: http://www.health.gov.za/index.php/antimicrobial-resistance

[B76] NDoH (2015c). Republic of South Africa. Essential Drugs Programme. Hospital level (Adults) Standard Treatment Guidelines and Essential Medicines List. 4th Edn. Republic of South Africa: National Department of Health Available online at: https://www.idealclinic.org.za/docs/guidelines/Hospital%20level%20(Adult)%202015.pdf

[B77] NDoH (2015d). National Policy for the Establishment and Functioning of Pharmaceutical and Therapeutics Committees in South Africa. Available online at: http://www.health.gov.za/index.php/pharmaceutical-and-therapeutics-committees

[B78] NDoH (2016). National Department of Health Republic of South Africa. Adherence Guidelines for HIV, TB and NCDs. Policy and Service Delivery Guidelines to Care, Adherence to Treatment and Retention in Care. February 2016. Available online at: https://www.nacosa.org.za/wp-content/uploads/2016/11/Integrated-Adherence-Guidelines-NDOH.pdf

[B79] NDoH (2017a). National Department of Health (NDoH), Statistics South Africa (Stats SA), South African Medical Research Council (SAMRC), and ICF. 2017. South Africa Demographic and Health Survey 2016: Key Indicators. Available online at: http://www.statssa.gov.za/publications/Report%2003-00-09/Report%2003-00-092016.pdf

[B80] NDoH (2017b). Government Gazette, 4 January 2017. Norms and Standards Regulations Applicable to Different Categories of Health Establishments. Available online at: http://www.samed.org.za/Filemanager/userfiles/national-health-act-61-2003-norms-and-standards-regulations-applicable-to-different-categories-of-health-establishments_20170104-GGN-40539-00010.pdf

[B81] NDoH (2017c). Government Gazette, 7 July 2017. NHI Implementation: Institutions, Bodies and Commissions that Must Be Established. Available online at: http://www.gov.za/sites/www.gov.za/files/40969_gon660.pdf

[B82] Nielsen J. O. Shrestha A. D. Neupane D. Kallestrup P. (2017). Non-adherence to anti-hypertensive medication in low- and middle-income countries: a systematic review and meta-analysis of 92443 subjects. J. Hum. Hypertens. 31, 14–21. 10.1038/jhh.2016.31 27306087

[B83] Norman C. Zarrinkoub R. Hasselstrom J. Godman B. Granath F. Wettermark B. (2009). Potential savings without compromising the quality of care. Int. J. Clin. Pract. 63, 1320–1326. 10.1111/j.1742-1241.2009.02129.x 19691615

[B84] O'Neill J. (2015). Securing New Drugs for Future Generations: The Pipeline of Antibiotics. The Review of Antimicrobial Resistance. Available online at: https://amr-review.org/sites/default/files/SECURING%20NEW%20DRUGS%20FOR%20FUTURE%20GENERATIONS%20FINAL%20WEB_0.pdf

[B85] Parkinson B. Sermet C. Clement F. Crausaz S. Godman B. Garner S.. (2015). Disinvestment and value-based purchasing strategies for pharmaceuticals: an international review. Pharmacoeconomics 33, 905–924. 10.1007/s40273-015-0293-8 26048353

[B86] Paruk F. Richards G. Scribante J. Bhagwanjee S. Mer M. Perrie H. (2012). Antibiotic prescription practices and their relationship to outcome in South Africa: findings of the prevalence of infection in South African intensive care units (PISA) study. S. Afr. Med. J. 102, 613–616. 10.7196/SAMJ.5833 22748439

[B87] Perumal-Pillay V. A. Suleman F. (2016). Quantitative evaluation of essential medicines lists: the South African case study. BMC Health Serv. Res. 16:687. 10.1186/s12913-016-1937-x 27955710 PMC5154091

[B88] Perumal-Pillay V. A. Suleman F. (2017a). Parents' and guardians' perceptions on availability and pricing of medicines and healthcare for children in eThekwini, South Africa - a qualitative study. BMC Health Serv. Res. 17:417. 10.1186/s12913-017-2385-y 28629443 PMC5477259

[B89] Perumal-Pillay V. A. Suleman F. (2017b). Selection of essential medicines for South Africa - an analysis of in-depth interviews with national essential medicines list committee members. BMC Health Serv. Res. 17:17. 10.1186/s12913-016-1946-9 28061899 PMC5219715

[B90] Pharasi B. Miot J. (2012). Medicines Selection and Procurement in South Africa. SAHR 2012/2013, 177–185.

[B91] Pillay-van Wyk V. Msemburi W. Laubscher R. Dorrington R. E. Groenewald P. Glass T.. (2016). Mortality trends and differentials in South Africa from 1997 to 2012: second National Burden of Disease Study. Lancet Global Health 4, e642–e653. 10.1016/S2214-109X(16)30113-9 27539806

[B92] Putrik P. Ramiro S. Kvien T. K. Sokka T. Pavlova M. Uhlig T.. (2014a). Inequities in access to biologic and synthetic DMARDs across 46 European countries. Ann. Rheum. Dis. 73, 198–206. 10.1136/annrheumdis-2012-202603 23467636

[B93] Putrik P. Ramiro S. Kvien T. K. Sokka T. Uhlig T. Boonen A. (2014b). Variations in criteria regulating treatment with reimbursed biologic DMARDs across European countries. Are differences related to country's wealth? Ann. Rheum. Dis. 73, 2010–2021. 10.1136/annrheumdis-2013-203819 23940213

[B94] Ramdas N. Meyer J. C. Cameron D. (2015). Factors associated with retention in HIV care at Sediba Hope Medical Centre. South. Afr. J. HIV Med. 16, 1–6. 10.4102/sajhivmed.v16i1.347 PMC5843141 29568580

[B95] Rampamba E. M. Meyer J. C. Helberg E. Godman B. (2017). Knowledge of hypertension and its management among hypertensive patients on chronic medicines at primary health care public sector facilities in South Africa; findings and implications. Expert Rev. Cardiovasc. Ther. 15, 639–647. 10.1080/14779072.2017.1356228 28712328

[B96] Republic of South Africa (2012). National Development Plan 2030: Our Future - Make It Work. Available online at: http://www.gov.za/issues/national-development-plan-2030

[B97] SAASP (2017). Mission Statement of the South African Antibiotic Stewardship Programme. Available online at: http://www.fidssa.co.za/SAASP

[B98] SAMRC (2016). Burden of Health & Disease in South Africa: Medical Research Council briefing. NCOP Social Services. Available online at: https://pmg.org.za/committee-meeting/22198/

[B99] Schellack N. Dlamini N. Meyer J. C. Godman B. (2017). Point Prevalence Survey of Antimicrobial Utilisation in an Academic Hospital in the Gauteng Province, South Africa. MURIA 3; 7 Available online at: http://muria.mandela.ac.za/muria/media/Store/documents/Abstract%20book%20-%20MURAI%203/MURIA3-AbstractBook-July-2017.pdf

[B100] Schellack N. Pretorius R. Messina A. P. (2016). Esprit de corps': Towards collaborative integration of pharmacists and nurses into antimicrobial stewardship programmes in South Africa. S. Afr. Med. J. 106, 973–974. 10.7196/SAMJ.2016.v106i10.11468 27725011

[B101] Sekhejane P. (2013). South African National Health Insurance (NHI) Policy: Prospects and Challenges for its Efficient Implementation. Policy Brief. Africa Institute of South Africa Briefing NO 102, December 2013. Available online at: http://www.ai.org.za/wp-content/uploads/downloads/2013/12/South-African-National-Health-Prospects-and-Challenges-for-its-Efficient-Implementation.pdf

[B102] Sooruth U. R. Sibiya M. Sokhela D. G. (2015). The use of Standard Treatment Guidelines and Essential Medicines List by professional nurses at primary healthcare clinics in the uMgungundlovu District in South Africa. Int. J. Afr. Nurs. Sci. 3, 50–55. 10.1016/j.ijans.2015.08.001

[B103] Statistics South Africa (2015). Mortality and causes of death in South Africa, 2014: Findings from death notification/Statistics South Africa. Pretoria. Available online at: http://www.statssa.gov.za/publications/P03093/P030932014.pdf

[B104] Statistics South Africa (2016). Mid Year Population Statistics 2016. Available online at: http://www.statssa.gov.za/publications/P0302/P03022016.pdf

[B105] Suleman F. (2010). Pharmacovigilance – who is responsible and why should we care? S. Afr. Pharm. J. 2010, 56–58.

[B106] Terblanche A. Meyer J. C. Godman B. Summers R. S. (2017a). Knowledge, attitudes and perspective on adverse drug reaction reporting in a public sector hospital in South Africa: baseline analysis. Hosp. Pract. [Epub ahead of print]. 10.1080/21548331.2017.1381013 28914115

[B107] Terblanche A. Meyer J. C. Summers R. S. Godman B. (2017b). Impact of a Pharmacist-Driven Pharmacovigilance System in a Secondary Hospital in the Gauteng Province of South Africa. MURIA 3; 25 Available online at: http://muria.mandela.ac.za/muria/media/Store/documents/Abstract%20book%20-%20MURAI%203/MURIA3-AbstractBook-July-2017.pdf 10.1080/21548331.2018.1510708 30092683

[B108] Toit J. du. (2017). Alternative models for delivery of medication to stable patients on long term therapy. S. Afr. Pharm. J. 84, 56–57.

[B109] Truter A. Schellack N. Meyer J. C. (2017). Identifying medication errors in the neonatal intensive care unit and paediatric wards using a medication error checklist at a tertiary academic hospital in Gauteng, South Africa. S. Afr. J. Child Health 11, 5–10. 10.7196/SAJCH.2017.v11i1.1101

[B110] Vagiri R. V. Meyer J. C. Gous A. G. S. (2015). Health-related quality of life and adherence to antiretroviral treatment over a 12-month period for patients attending two public sector clinics in South Africa. Afr. J. Phys. Health Educ. Recreation Dance Suppl. 2.2, 228–240.

[B111] Wang H. Naghavi M. Allen C. Barber R. M. Bhutta Z. A. Carter A. (2016a). Global, regional, and national life expectancy, all-cause mortality, and cause-specific mortality for 249 causes of death, 1980-2015: a systematic analysis for the Global Burden of Disease Study 2015. Lancet 388, 1459–1544. 10.1016/S0140-6736(16)31012-1 27733281 PMC5388903

[B112] Wang H. Wolock T. M. Carter A. Nguyen G. Kyu H. H. Gakidou E. (2016b). Estimates of global, regional, and national incidence, prevalence, and mortality of HIV, 1980-2015: the Global Burden of Disease Study 2015. Lancet HIV 3, e361–e387. 10.1016/S2352-3018(16)30087-X 27470028 PMC5056319

[B113] Ware N. C. Idoko J. Kaaya S. Biraro I. A. Wyatt M. A. Agbaji O.. (2009). Explaining adherence success in sub-Saharan Africa: an ethnographic study. PLoS Med. 6:e11. 10.1371/journal.pmed.1000011 19175285 PMC2631046

[B114] WHO (2013). A Global Brief on Hypertension - Silent Killer, Global Public Health Crisis. World Health Day 2013. Available online at: http://apps.who.int/iris/bitstream/10665/79059/1/WHO_DCO_WHD_2013.2_eng.pdf?ua=1

[B115] WHO (2014). Antimicrobial Resistance. Global Report on Surveillance. Available online at: http://www.euro.who.int/en/health-topics/disease-prevention/antimicrobial-resistance/news/news/2014/04/new-report-antibiotic-resistance-a-global-health-threat

[B116] WHO (2015). Lobal Action Plan on Antimicrobial Resistance. Available online at: http://www.who.int/antimicrobial-resistance/publications/global-action-plan/en/

